# A Deep Q-Network based hand gesture recognition system for control of robotic platforms

**DOI:** 10.1038/s41598-023-34540-x

**Published:** 2023-05-17

**Authors:** Patricio J. Cruz, Juan Pablo Vásconez, Ricardo Romero, Alex Chico, Marco E. Benalcázar, Robin Álvarez, Lorena Isabel Barona López, Ángel Leonardo Valdivieso Caraguay

**Affiliations:** 1 Artificial Intelligence and Computer Vision Research Lab, Departamento de Informática y Ciencias de la Computación (DICC), Escuela Politécnica Nacional, Ladrón de Guevara, 170517 Quito, Ecuador; 2 Departamento de Automatización y Control Industrial (DACI), Escuela Politécnica Nacional, Ladrón de Guevara, 170517 Quito, Ecuador; 3 Faculty of Engineering, Universidad Andres Bello, Antonio Varas, Santiago, Chile

**Keywords:** Energy science and technology, Engineering

## Abstract

Hand gesture recognition (HGR) based on electromyography signals (EMGs) and inertial measurement unit signals (IMUs) has been investigated for human-machine applications in the last few years. The information obtained from the HGR systems has the potential to be helpful to control machines such as video games, vehicles, and even robots. Therefore, the key idea of the HGR system is to identify the moment in which a hand gesture was performed and it’s class. Several human-machine state-of-the-art approaches use supervised machine learning (ML) techniques for the HGR system. However, the use of reinforcement learning (RL) approaches to build HGR systems for human-machine interfaces is still an open problem. This work presents a reinforcement learning (RL) approach to classify EMG-IMU signals obtained using a Myo Armband sensor. For this, we create an agent based on the Deep Q-learning algorithm (DQN) to learn a policy from online experiences to classify EMG-IMU signals. The HGR proposed system accuracy reaches up to $97.45 \pm 1.02\%$ and $88.05 \pm 3.10\%$ for classification and recognition respectively, with an average inference time per window observation of 20 ms. and we also demonstrate that our method outperforms other approaches in the literature. Then, we test the HGR system to control two different robotic platforms. The first is a three-degrees-of-freedom (DOF) tandem helicopter test bench, and the second is a virtual six-degree-of-freedom (DOF) UR5 robot. We employ the designed hand gesture recognition (HGR) system and the inertial measurement unit (IMU) integrated into the Myo sensor to command and control the motion of both platforms. The movement of the helicopter test bench and the UR5 robot is controlled under a PID controller scheme. Experimental results show the effectiveness of using the proposed HGR system based on DQN for controlling both platforms with a fast and accurate response.

## Introduction

Conventional human-machine interfaces (HMIs) have been proposed to control robotic platforms by using different devices such as keyboards, joysticks, inertial measurement units (IMUs)^1^, vision-based systems^2,3^, haptic devices^4^, and speech recognition systems^5^. However, in the last years, the use of non-verbal communication techniques has been demonstrated to be useful when creating human-machine interfaces (HMIs). In particular, hand gesture recognition (HGR) systems have been used in applications such as sign language recognition, muscle rehabilitation systems, prostheses, robotics, augmented reality, and image manipulation, among others^6^. However, the control of robotic platforms that uses HGR systems is still an open research problem^1,7^. These HGR systems can be divided into vision-based and sensor-based depending on the sensor that is used. Several HGR systems use vision-based methods, such as the Kinect^8^ and the Leap Motion Sensor^9^. However, its functionality can be affected by occlusion problems and the distance between the sensor and the hand. On the contrary, sensor-based HGR systems use gloves with inertial measurement units^10,11^, as well as surface electromyography (EMG) non-invasive methods for detection of arm muscle activity such as G-Force and Myo Armband sensors^12^. In general, no matter what type of sensor is used, designing HGR systems that are able to determine the moment in which a certain hand gesture was executed is challenging. This is in part due to the variability of the signals of each gesture between different users, as well as the similarities that are present between different gestures for the same user^7^. However, the design of HGR systems is important as these systems can help to increase the performance, accuracy, and versatility of teleoperation tasks to control robotic platforms.

Several applications have previously used HGR systems to control robotic platforms. Most of these works developed HMIs to control different kinds of robots by using EMG and IMU measures, as well as vision sensors. For example, the authors in^13^ designed a HMI to control an underwater robot by using EMG signals, a gyroscope, and a camera. They used a fuzzy-PID algorithm to command the underwater robot postures. Other work designed an architecture that used upper limb motion estimation based on EMG signals and a Kinect sensor to control a humanoid robotic arm^14^. Another work proposed a HMI that used the orientation and muscle force of the forearm, as well as dynamic hand motions to control the position and configurations of a six degrees of freedom (DoF) robotic manipulator with one DoF gripper^15^. The proposed system was able to recognize dynamic hand motions by using EMG and IMU sensors to control the robot in real-time. In^1^, the authors propose an EMG-based HGR system based on two Myo Armband devices to track the movement of both arms of a user to command a three DoF commercial manipulator. For this, a non-linear robust control was proposed to tackle the tracking problem. The use of EMG-based HGR systems has been also studied to control unmanned aerial vehicles (UAVs). For example, in^16,17^, the authors propose an EMG-based HGR system to control drones through hand gestures in a natural and intuitive manner without the need for a joystick or other interface. Another work presents an architecture based on Convolutional Neural Networks (CNN) and Recurrent Neural Networks (RNN) to design an EMG-based HGR system that uses the Myo Armband sensor to command a virtual quadcopter with Dronekit^18^. Finally, a multi-modal UAV control was proposed in^17^, which uses an EMG-IMU-based HGR system for controlling quadcopters with an 81.5% gesture recognition accuracy. The proposed HGR system was based on a CNN that was used to learn hand gestures and movement patterns.

As we presented in the previous paragraph, several methods have been developed based on EMG and/or IMU to command the movement and operation modes of various robotic platforms. However, it is important to mention that each robotic platform could present a different behavior and requirements. In addition, each user can have a different response to the HGR sensors and can behave differently when using the robotic platforms. Therefore, it is important to investigate different HGR models to evaluate them on different robotic platforms. In general, machine learning (ML) and deep learning (DL) techniques have been previously used to develop EMG or EMG-IMU-based HGR systems^19,20^. In particular, supervised methods such as decision trees, support vector machines (SVM), artificial neural networks (ANN), convolutional neural networks (CNN), and recurrent neural networks (RNNs) have shown high accuracy for HGR systems^19,21^. However, conventional ML and DL models still require a fully labeled dataset with all the user’s features and labels to be trained, which makes it difficult to learn from the online experience. This makes online learning difficult, making it not viable when the HGR system needs to be trained while the user is interacting with the HGR system. On the other hand, reinforcement learning (RL) approaches can be used to build models that learn from experience online. It is in this context where the concept of adaptability comes into play since an HGR system based on EMG-IMU signals should be able to present certain adaptability to users over time. There have been a few attempts to use RL methods to develop HGR systems. For example, in^22^, the authors used the UCI dataset where six subjects realized different hand movements. To learn a classification policy, a dueling deep Q-learning technique was used. This approach learns to classify between 6 hand gestures. In^23^, a reinforcement learning-based classifier for arm and finger movement was presented, where a 26TSystem was used to obtain EMG signals from 10 subjects. A Q-learning based on a feed-forward neural network classifier was used to infer six classes of elbow positions and 4 finger movement classes. The authors indicate that the proposed method based on Q-learning outperforms supervised methods for their dataset distribution. Finally, we have developed architectures that work with reinforcement learning algorithms such as Q-learning to develop HGR systems using only EMGs to recognize 6 hand gestures^24^. The proposed model required a large amount of data and training time, as well as a very sensitive and extensive calibration of their hyper-parameters to obtain promising results^24^. As explained in these works, the use of RL algorithms can be used to obtain high-performance EMG-based HGR systems. However, although the results obtained when using EMG and reinforcement learning to recognize hand gestures were encouraging, it is still necessary to explore other types of algorithms and sensor information, such as the combination of EMG-IMU signals with agents based on convolutional neural networks (CNN). Furthermore, the use of these methods in simulated and real robotic platforms also needs to be rigorously evaluated.

Based on this information, related to reinforcement learning algorithms for hand gesture recognition for robotic platforms control, we can say the following. First, the databases that have been used to date have been proposed by each author, and data is not always available online. Moreover, the combination of EMG-IMU signals for the development of HGR systems based on reinforcement learning is still an open research topic. Finally, to date, we have not found a work that uses HGR systems based on RL methods to compare control systems for both a built robotic platform and a robot in a simulator, which is one of the main motivations of this work.

In this context, we propose the use of a reinforcement learning algorithm that uses EMG-IMU signals to address the problem of HGR systems to control different robotic platforms.

The main contributions of this work are summarized as follows:

- We successfully developed an EMG-IMU-based HGR system that uses a reinforcement learning approach based on a Deep Q-network (DQN) to recognize 6 different hand gestures (5 gestures and 1 no gesture). The algorithm was trained and tested by using a dataset composed of 32 users (16 for training and 16 for testing).
- We assess the effectiveness of the EMG-IMU-based HGR system and the IMU signals to control a 3-DOF tandem helicopter test-bench. For this, a position controller based on a cascade PID structure has been tested to control this platform.
- We assess the effectiveness of the EMG-IMU-based HGR system and the IMU signals to control a 6-DOF UR5 virtual manipulator in a virtual environment. For this, a minimum norm PID controller has been tested to control this platform.

## Materials and methods

**Figure 1:**
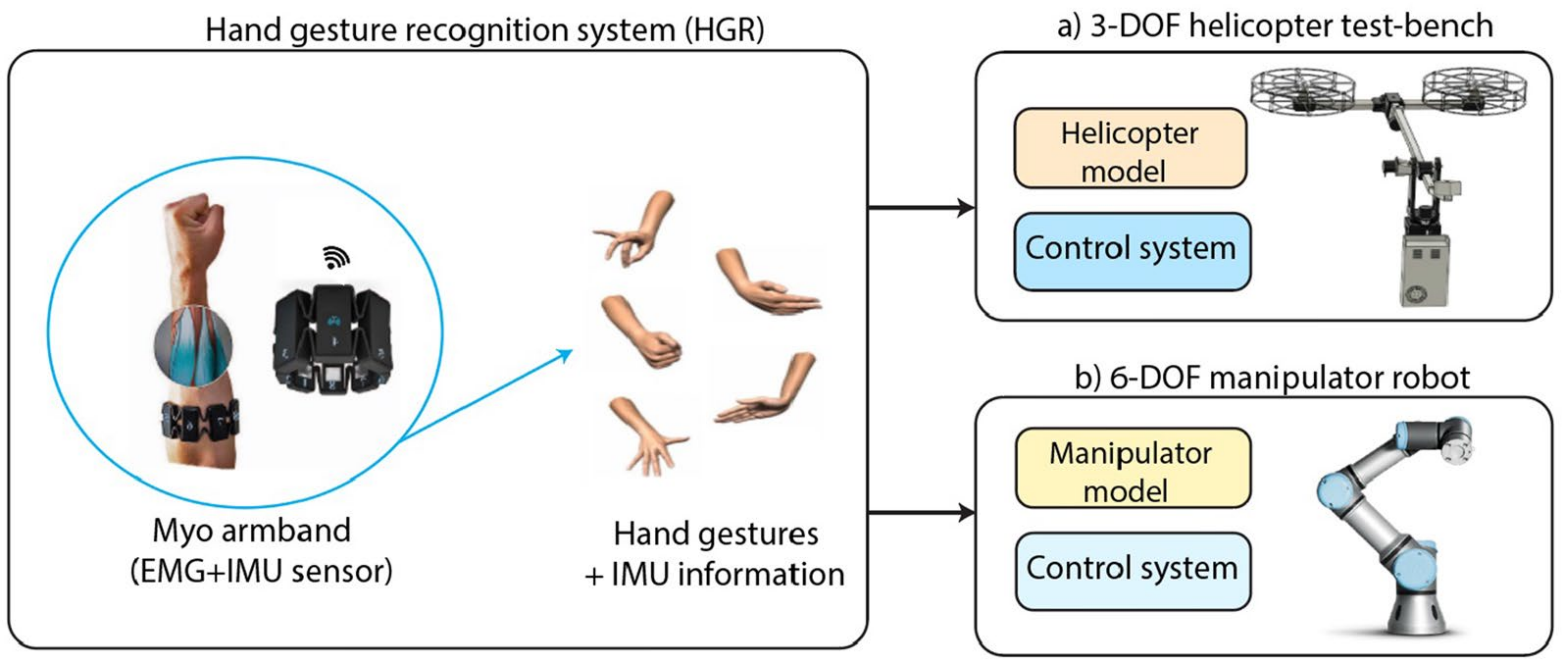
Proposed architecture based on a HGR system to control both a 3-DOF tandem helicopter test-bench and a 6-DOF UR5 virtual manipulator^25,26^.

In this section, we present the proposed architecture based on a HGR system to control both a 3-DOF tandem helicopter test-bench and a 6-DOF UR5 virtual manipulator, which is illustrated in Fig. 1. As can be observed, the proposed architecture is conformed by the HGR system, the 3-DOF tandem helicopter test-bench, and the 6-DOF UR5 virtual manipulator. We explain in detail each stage of the proposed architecture as follows.

### Hand gesture recognition system (HGR)

**Figure 2:**
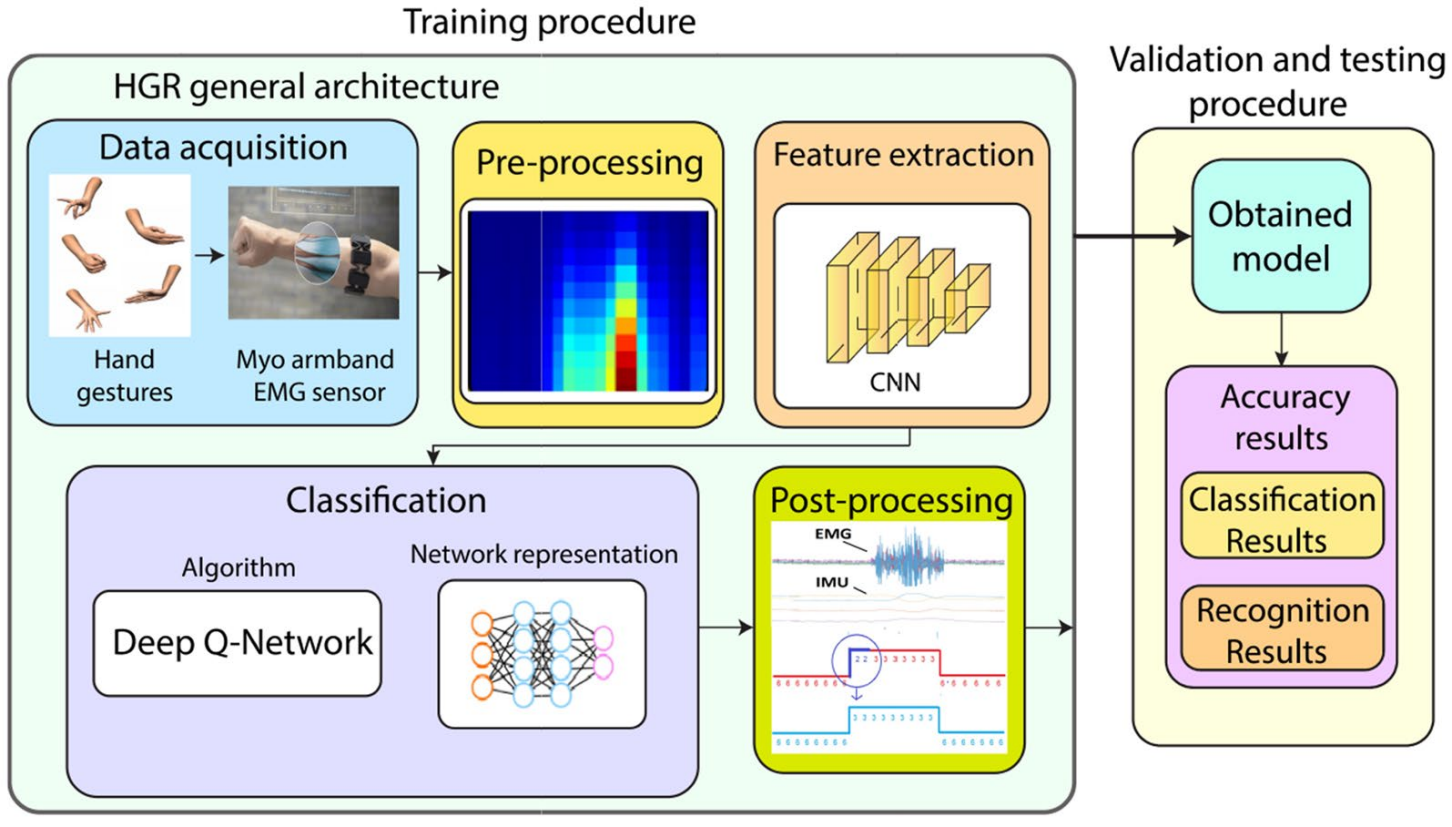
Hand gesture recognition (HGR) architecture based on EMG-IMU signals and DQN.

In this subsection, we present the proposed HGR system based on EMG-IMU signals and DQN as a RL-based method, which is illustrated in Fig. 2. As can be observed, the proposed architecture is conformed by data acquisition, pre-processing, feature extraction, classification, and post-processing stages. We explain in detail each stage of the HGR system as follows.

#### Data acquisition

We use EMG-IMU data of 5 different hand gestures—wave in, wave out, fist, open, pinch—and the no gesture or relax gesture—6 gestures in total—, which was collected by using the Myo armband device—8 channels at 200 Hz. The dataset has been made publicly available in the following link^27^. The dataset distribution is presented in Table 1. In this work, we trained and evaluated user-specific models, which means that each user information is trained with a single model.

**Table 1:** Data set distribution to evaluate user-specific models (one model for each of the 32 users).

	User-specific model			
	Models	Training	Validation	Test
Training set	16 models	150 samples per user	150 samples per user	–
Testing set	16 models	150 samples per user	–	150 samples per user

#### Pre-processing

For each EMG-IMU sample, we first perform a segmentation procedure that split the EMG-IMU signal into multiple windows to analyze one by one. For this, we use a sliding window approach to slide over the entire EMG-IMU signal^7,19,20^. To perform the segmentation procedure, we split a EMG-IMU signal sample of 5 seconds (1000 points) into multiple windows by using a sliding window of width $W=300$ points and a $stride=40$ (separation between windows). We select the window and stride values experimentally as a designer criterion since it helps us to reach high-accuracy results. For a given instant of time, we obtain measures from the Myo Armband sensor, where each component of such measure represents the EMG-IMU information for each channel $i=1,2,\ldots ,12$ respectively (8 channels for EMG and 4 for IMU signals).

Then, we perform a rectification procedure on the EMG-IMU signal by applying the absolute value calculation to avoid the mean in each channel being zero. In addition, we apply a low-pass Butterworth filter to the EMG-IMU signal to reduce noise. The filter has an order of 4 and a cut-off frequency of 15Hz to obtain a balance between the number of spectrograms and their dimensions.

To obtain relevant features from the EMG-IMU signals, we use the time-frequency domain, since more useful information can be obtained using this approach than by using only the time or frequency domains^12,28^. For this, once we obtained the filtered and rectified signal, we use the Short-time Fourier transform (STFT) by using an internal sliding window—width $=24$ points and stride $=12$ points—on each window observation. Finally, the spectrogram is calculated as the squared magnitude of the real and imaginary components of the STFT. It is to be noticed that the spectrogram is calculated for each of the 12 channels of the sensor, which includes EMG and IMU information. Finally, we concatenate the spectrogram of each channel to build a tensor which will be the input of the feature extraction stage.

#### Feature extraction

**Figure 3:**
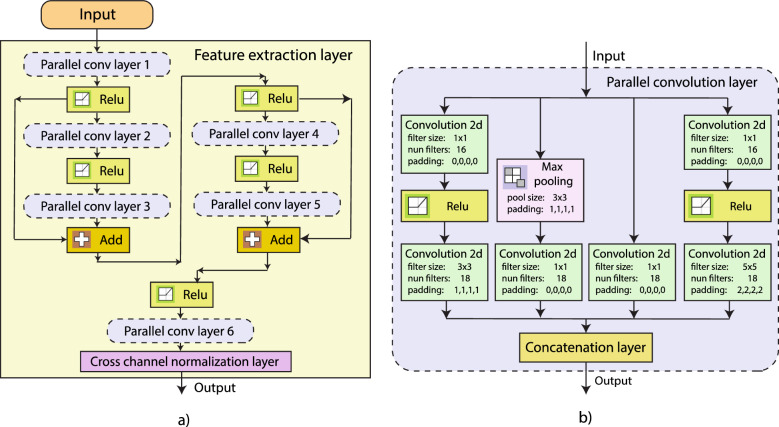
Feature extraction stage. (**a**) Feature extraction layer structure composed of residual blocks and parallel convolution layers. (**b**) Parallel convolution layer structure.

In this work, we use feature extraction methods to obtain relevant and non-redundant information from the EMG-IMU signal. For this, we used the spectrograms of the EMG-IMU signals (time-frequency domain features) to feed a CNN, which is known as a high-efficiency automatic feature extraction method. The proposed feature extraction stage uses residual blocks as can be observed presented in Fig. 3a, which helps to avoid the vanishing or exploding gradients problems and accelerates the training procedure^29^. The proposed feature extraction stage is composed of parallel convolution layers, which are built on several blocks of convolutions and max-pooling layers distributed in parallel, that are inspired by the Inception modules as illustrated in Fig. 3b^30^. The internal blocks of the parallel convolution layer allow the network to extract features with different convolution filter sizes, which makes possible the extraction of a wide number of features that help to achieve high classification performances. We used in total 6 parallel convolution layer structures and 2 residual blocks to build the feature extraction stage illustrated in Fig. 3a.

#### Classification

**Figure 4:**
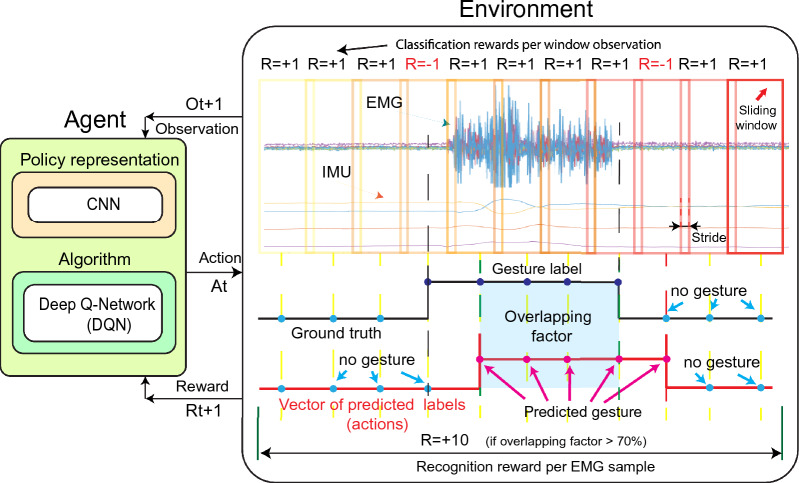
Illustration of the interaction between the DQN agent representation and the proposed environment for EMG-IMU signals classification and recognition.

The classification stage is used to infer the hand gesture category from the feature extraction stage information. For this, we define the EMG-IMU signals classification problem that uses the sliding window as a sequential decision problem, in which the prediction of the hand gestures represents the actions of an agent, and the EMG-IMU signals extracted features for each sliding window represents the observations^24,31^. Within this scheme, an optimal policy can be calculated from the optimal values by choosing the highest-valued action at each observation. In this work, we learn the estimates for the optimal action values by using the Deep Q-network (DQN) off-policy algorithm as illustrated in Fig. 4. As can be observed, our agent is composed of CNN as the policy representation, as well as the DQN algorithm to learn a policy that maximizes the total sum of rewards.

The proposed CNN-based agent learns to classify the labels of sliding windows that provide observations from the EMG-IMU signals. It is worth mentioning that we used the DQN algorithm since the EMG-IMU spectrogram tensors represent a continuous observation space, and an artificial neural network-based policy representation is recommended to build the agent^32^. Moreover, we use a critic agent representation since it usually obtains high-performance results for discrete action spaces, which in our case is represented by the 6 gesture categories^32^. Our critic agent infers the expected value of the long-term reward for a particular observation and action^32^. Hence, after taking action $A_{ t }$ in the observation $O_{ t }$ and earning the reward $R_{t+1}$ in $O_{t+1}$, the DQN algorithm adjusts the neural network parameters $\theta _{t}$, as detailed in the following expression:

1 $$\begin{aligned} {{ \theta _{ t+1 } } = { \theta _{ t } } + \alpha \left( Y^{DQN}_{t} - { Q }\left( { O }_{ t },{ A }_{ t }; { \theta _{ t } } \right) \right) \cdot \nabla _{\theta _{t} } { Q }\left( { O }_{ t },{ A }_{ t }; { \theta _{ t } } \right) } \end{aligned}$$

Where $\theta _{ t+1 }$ represents the updated learned parameters, and $\theta _{ t }$ represents the previous learned parameters respectively. The value of $\alpha$ represents the learning rate.

The DQN algorithm returns a set of action values for a certain observation ${ O }_{ t }$, where $Q({ O }_{ t }, \cdot ; \theta )$ denotes the parameters of the CNN as explained in^23,31,32^. The DQN algorithm uses a target network $Y^{DQN}_{t}$ from Eq. (2), which is defined as follows.

2 $$\begin{aligned} {Y^{DQN}_{t} \equiv { R }_{ t+1 } + \gamma \cdot \underset{ { a } }{ \max } \left[ { Q }\left( { O }_{ t+1 },{ a }, { \theta ^{-}_{t}} \right) \right] } \end{aligned}$$

where ${ R }_{ t+1 }$ represents the reward earned when going from the observation ${ O }_{ t }$ to the observation ${ O }_{ t+1 }$ by taking the action ${ A }_{ t }$. The optimal future Q value that is estimated is represented by the expression $\underset{ { a } }{ \max } [{ Q }\left( { O }_{ t+1 },{ a } \right) ]$, and the discount factor is denoted by $\gamma$.

The target network $Y^{DQN}_{t}$ from Eq. (2) contains parameters $\theta ^{-}$ that are updated regularly every $\tau$ steps from the online network in Eq. (1) which has the parameters $\theta _{t}$. Therefore, the parameters $\theta ^{-}$ are fixed for the remainder of the period until the subsequent update after $\tau$ steps. By doing this, correlations with the target are reduced^31,33^. Finally, we use experience replay to shuffle the data at random in order to eliminate correlations in the observation sequences, which helps to improve the accuracy of the DQN algorithm^31,33^.

Next, we explain in detail each element of the proposed scheme shown in Fig. 4 for the EMG-IMU sequential classification problem solved by DQN to learn an optimal policy:

- *Agent* it is composed of a CNN policy representation that can take action at any observation of the environment, and it is meant to learn a policy that maximizes the total sum of rewards by using the DQN algorithm. The proposed agent learns to classify the labels of the sliding windows from the observations of the EMG-IMU signals.
- *Observation* An observation is composed of part of the information of the real state of the environment with which the agent interacts. We define the observation ${ O }_{ t }$ as the tensor resulting from the feature extraction stage for each EMG-IMU sliding window respectively.
- *Action* The agent performs an action ${ A }_{ t }$ when is in the current observation ${ O }_{ t }$ to reach the observation ${ O }_{ t+1 }$ and then receive the reward ${ R }_{ t+1 }$. We define an agent action as one of the possible class gestures (wave in, wave out, fist, open, pinch, and no gesture).
- *Environment* We define the environment as the sliding window information—feature vectors and labels– extracted from the ground-truth of each EMG-IMU signal.
- *Reward* The agent receives a positive or negative reward from its interaction with the environment. We define two different rewards to learn the classification and recognition procedure (see Fig. 4). The agent can receive a positive reward ${ R }_{ t }=+1$ or a negative reward ${ R }_{ t }=-1$ depending if it predicts a window label correctly or incorrectly respectively. On the other hand, once an episode is over, the ground truth is compared with the predicted labels, and if the overlapping factor of the predicted gestures is more than 70%, then the recognition is considered successful and receives a positive reward ${ R }_{ t }=+10$.

#### Post-processing

We use a post-processing stage to eliminate erroneous labels from the predictions in the EMG-IMU signals. We calculate the mode on the predicted vector of classes that are different from the no-gesture label^24^. Then, all the labels in such vectors that are different from the mode gesture are replaced with such gesture for that EMG-IMU sample. This stage is important to improve recognition performance.

### 3-DOF tandem helicopter test-bench

**Figure 5:**
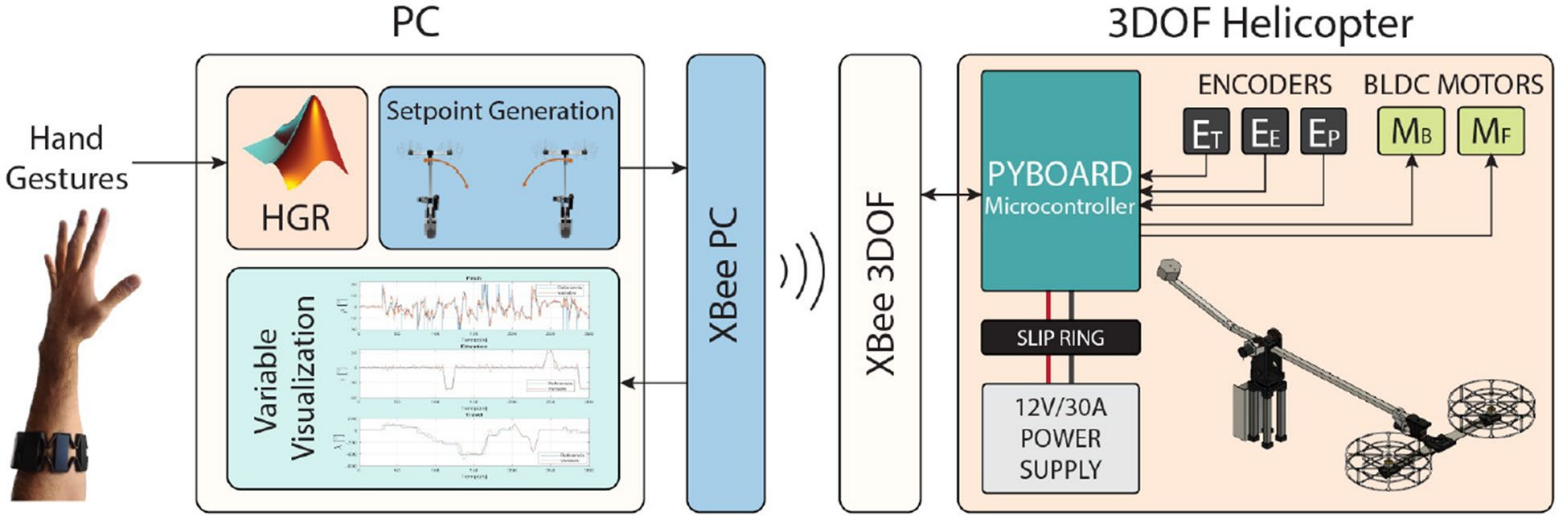
Architecture of the 3-DOF Helicopter controlled with the HGR system^25,26^.

The 3-DOF tandem helicopter test-bench is a simplified experimental platform for validating the effectiveness of different control systems for unmanned aerial vehicles. In this work, we used the 3-DOF helicopter designed by using low-cost materials, 3D printing, and laser cutting presented in^34^. This test-bench receives its name from its 3 motion angles, which are called *travel, elevation* and *pitch*. The hand gesture recognition system (HGR) described in previous sections is used to generate setpoint values that are then transmitted to the helicopter. Internally, the 3-DOF helicopter has a position controller which is responsible for the motion along the three axes. The proposed architecture for controlling the test-bench is presented in Fig. 5. As it is shown in this figure, the system is composed of two main components which are the PC and the helicopter itself. We explain in detail each stage of the HGR system as follows.

**Figure 6:**
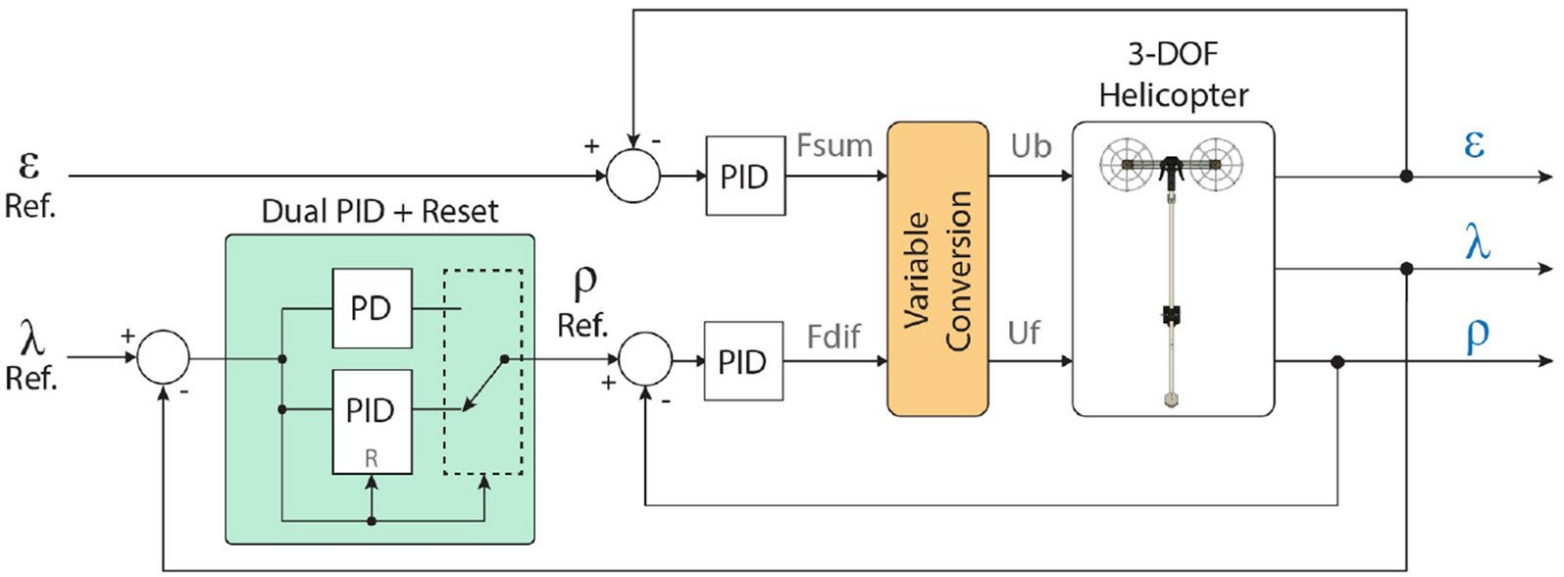
PID controller scheme implemented for orientation control of the 3-DOF Helicopter.

- *PC* all the signals acquired from the Myo Armband sensor are interpreted, and a set point command is generated according to the gesture performed by the user. The PC is also used as an interface for the user to visualize and interact with the variables of the helicopter.
- *3-DOF Helicopter* The prototype has an embedded PID control system inside a Pyboard microcontroller which receives the set point value from the PC through an XBee module. The feedback controller is implemented using three incremental encoders as measurement devices (one for each angle of motion: ${E_T, E_E, E_P}$ for the variables travel, elevation and pitch, respectively). The control laws are calculated (based on the control diagram shown in Fig. 6 and Eqs. (8)–(10)) and then transformed into a Pulse Width Modulation signal (PWM) which is then sent to the brushless DC motors (BLDC, represented by ${M_F}$ and ${M_B}$ for the front and back motors in Fig. 5) that generate all the motion of the helicopter. The prototype is powered by a 12V/30A supply whose power is transmitted through a slip ring, which is needed to avoid cables from tangling by the rotation of the helicopter about the travel axis.

#### Helicopter model

The main structure of the 3-DOF helicopter is made of aluminum profiles and the supporting structures for each rotation axis and encoders were 3D printed. A more detailed explanation of the physical construction of the Helicopter test-bench is given by^34^.

To obtain a mathematical model of the motion of the 3-DOF helicopter it is important to analyze its dynamics, which are mainly given by its geometry. The masses of the different components, and the two input forces, which correspond to the thrust generated by each propeller, are called $F_{b}$, $F_{f}$ for the back and front motors, respectively. These two forces are the ones that make the helicopter move along its three axes of motion: travel $\lambda$, elevation $\epsilon$, and pitch $\rho$. These 3 variables are the outputs of the system and can be related to the input forces as follows: when the two forces, $F_{b}$ and $F_{f}$ are equal to each other and different from zero, a total thrust force $F_{sum} = F_b + F_f$ will appear in the direction of motion of the elevation, $\epsilon$ axis. Similarly, when the two forces are different from each other, the difference $F_{dif} = F_f - F_b$ will accelerate the helicopter body in the direction of the pitch $\rho$ axis. Since these compound forces $F_{sum}$ and $F_{dif}$ can describe the motion of the helicopter in a better way, they are used for the derivation of the mathematical model. In order to implement a control law that uses these new forces, it is important to consider the transformation between each propeller force ($F_{b}$ and $F_{f}$) into $F_{sum}$ and $F_{dif}$, which is shown in Eq. (3).

3 $$\begin{aligned} F_f = \frac{F_{sum}+F_{dif}}{2} \quad \text {and} \quad F_b = \frac{F_{sum}-F_{dif}}{2} \end{aligned}$$

Lastly, the motion of the prototype about the travel $\lambda$ axis is dependent on the angle $\rho$. When a positive pitch appears, the force $F_{sum}$ pushes the helicopter in the positive direction of the $\lambda$ axis, and the opposite is also true. Therefore, it can be noted that the pitch motion and the travel motion are strongly coupled and dependent on each other. From this analysis and the measurements of the different lengths and masses of the system, a set of mathematical equations that describe the motion of the 3-DOF helicopter can be derived as shown in the Eqs. (4)–(6)^35^.

4 $$\begin{aligned} \ddot{\rho } I_{\rho }&= L_{H} F_{dif} \end{aligned}$$

5 $$\begin{aligned} \ddot{\epsilon } I_{\epsilon }&= L_{M} \cos {(\rho )} F_{sum} - \left( L_{M}m_{H}-L_{W}m_{W} \right) \cos {(\epsilon )} \end{aligned}$$

6 $$\begin{aligned} \ddot{\lambda } I_{\lambda }&= L_{M} \cos {(\epsilon )} \sin {(\rho )} F_{sum} \end{aligned}$$

where $I_{\lambda }$, $I_{\epsilon }$, and $I_{\rho }$ are the moments of inertia about the travel, elevation and pitch axes respectively. $L_{M}$ is the distance from the travel axis to the helicopter body, $L_{H}$ is the length measured from the pitch axis to each motor, and $L_{W}$ is the distance from the travel axis to the counterweight, whose mass is represented by $m_{W}$. The body of the helicopter has a total mass of $m_{H}$, and the mass of each motor-propeller assembly is represented by $m_{h}$.

#### Helicopter control system

From the equations of motion for the 3-DOF helicopter described above, we can design a control system for regulating the outputs of the system. For this 3-DOF helicopter, a PID controller was designed for each variable. The implemented control scheme is shown in Fig. 6. The relations between variables and design considerations for each controller are explained as follows:

- *Pitch* as it was explained earlier, when the propeller forces $F_{b}$ and $F_{f}$ are different from each other, the value of $F_{dif}$ will have a value other than 0, and a torque will appear around the pitch axis. The PID controller for this variable measures the value of $\rho$ and generates a corresponding PWM signal for the motors, which in turn produces a $F_{dif}$ force to reach the set point $\rho _{ref}$.
- *Elevation* similar to the pitch axis, when the two propellers are moving, a net thrust $F_{sum}$ will appear, which will accelerate the helicopter in the $\epsilon$ direction. In this case, the PID controller has the error of the elevation axis as input and the output is a PWM value that generates the thrust along the elevation axis.
- *Travel* as it was explained in previous paragraphs, when the pitch angle is different than 0, a component of the net thrust appears in the travel direction and is equal to $F_{sum} \sin {(\rho )}$ which is why for this variable the input is considered to be the angle $\rho$. This behavior creates a cascade control loop that has a travel controller on the outside and the pitch controller becomes the inner loop. Therefore, in this case, the PID controller generates a setpoint value for the $\rho$ angle.

In addition to these features, the performance of the travel controller was improved by adding a reset on the integral action and a cascade PD-PID structure. This feature is used to accomplish two important improvements to the travel motion:

- *Reduced overshoot* the reset is applied when the error is less than $3^{\circ }$. This threshold was implemented considering that the span of the travel motion is much larger. Also, the surrounding air produces a disturbance due to turbulence, whose effect lies within the range of $\pm 3^{\circ }$. With this condition, the overshoot of the travel motion is effectively reduced because the integral action is reset every time the error $e_\lambda$ is in that range.
- *Anti-windup* the second reset condition is applied when the error is greater than $20^{\circ }$, outside of that range only a PD controller is applied. This is used to counteract the effect of excessive integral windup, which happens when a large change in the set point value is sent to the helicopter. The $20^{\circ }$ was determined empirically after several tests in which it was observed that the PD controller alone was capable of achieving an error below $17^{\circ }$.

This dual PD-PID characteristic was implemented as an interval of travel error in which the integral action is applied. This interval is represented by $\Delta _{e_\lambda }$ and is defined in Eq. (7).

7 $$\begin{aligned} \Delta e_\lambda = e_\lambda (t) \ge \mid 3^\circ \mid \wedge \, e_\lambda (t) < \mid 20^\circ \mid \end{aligned}$$

The full control scheme with the additional features mentioned above is shown in Fig. 6. As it can be noticed, when the error $e_\lambda$ lies within the specified range given by $\Delta _{e_\lambda }$ , the controller switches from a PID structure to PD or vice-versa.

Additionally, the control actions for each variable are represented by Eqs. (8)–(10). Here it is easier to see the relationship between the travel variable, $\lambda$ and the pitch variable $\rho$. The output of the $\lambda$ controller is the reference input (or setpoint) for the $\rho$ control loop, as it was previously explained. In these equations, $K_p, K_i$ and $K_d$ represent the proportional, integral and derivative gains for the PID controller for each variable. Each gain also has a subscript for each variable. All of these gains were obtained through empirical methods as it is discussed in^34^. It can also be observed in Eqs. (8)–(10) that the elevation variable is related to the sum of the motor thrust forces or $F_{sum}$ and the pitch variable changes accordingly with the difference of thrusts, $F_{dif}$

8 $$\begin{aligned} \rho _{ref}(t)&= \left\{ \begin{array}{lcc} Kp_\lambda e_\lambda (t) + Ki_\lambda \int e_\lambda (t) dt + Kd_\lambda \frac{de_\lambda (t)}{dt} &{} if &{} e_\lambda (t) \in \Delta e_\lambda \\ Kp_\lambda e_\lambda (t) + Kd_\lambda \frac{de_\lambda (t)}{dt} &{} if &{} e_\lambda (t) \notin \Delta e_\lambda \end{array} \right. \end{aligned}$$

9 $$\begin{aligned} F_{dif}(t)&= \begin{array}{lcc} Kp_\rho e_\rho (t) + Ki_\rho \int e_\rho (t) dt + Kd_\rho \frac{de_\rho (t)}{dt} ,&e_\rho (t) = \rho _{ref}(t) - \rho (t) \end{array} \end{aligned}$$

10 $$\begin{aligned} F_{sum}(t)&= Kp_\epsilon e_\epsilon (t) + Ki_\epsilon \int e_\epsilon (t) dt + Kd_\epsilon \frac{de_\epsilon (t)}{dt} \end{aligned}$$

#### HGR for the 3-DOF helicopter

**Figure 7:**
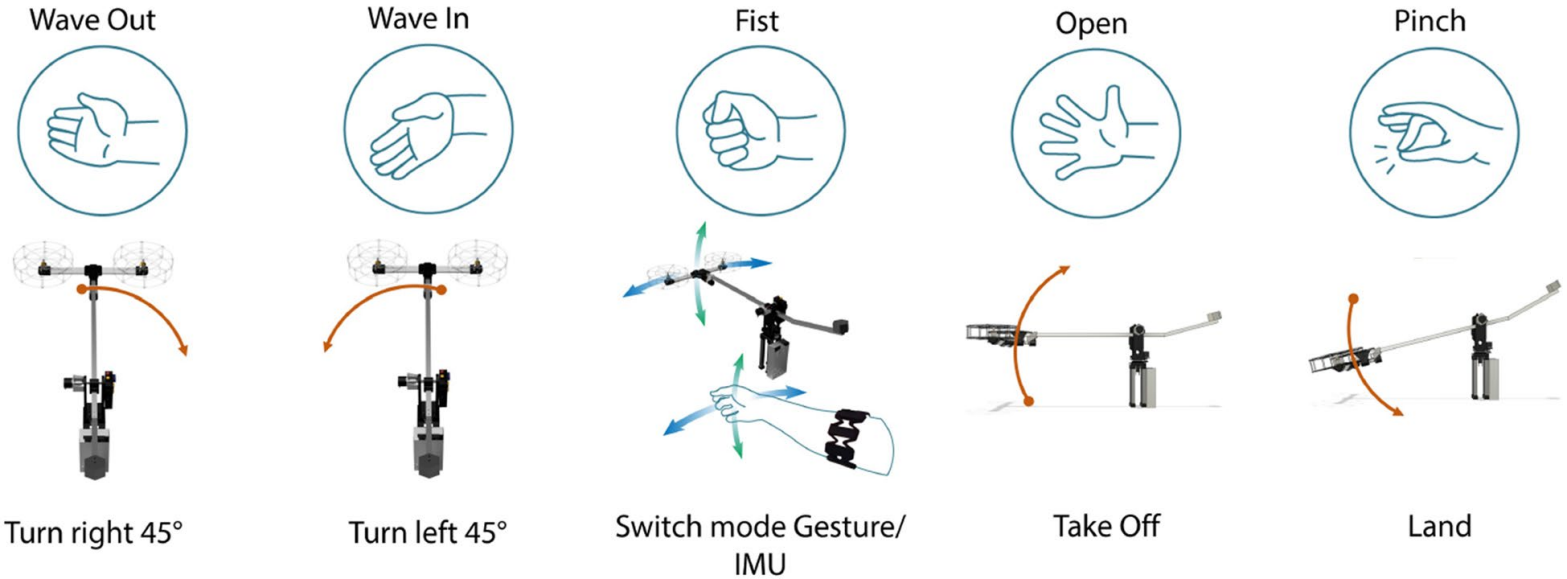
Different flight commands for the 3-DOF Helicopter according to each gesture. The *fist* gesture is used to switch between gesture and IMU modes.

The HGR system was integrated with the 3-DOF helicopter test-bench so that each gesture produces a different flight command. An additional feature that has been implemented to the motion controller of the helicopter is the ability to control with two modes: a hand gesture mode and an arm motion (IMU) mode, which are explained below. Each of the five gestures (wave out, wave in, fist, open, and pinch) corresponds to a different action that the helicopter can perform as it is specified in Fig. 7.

- *Gesture mode* in this mode, each of the four gestures Wave Out, Wave In, Open and Pinch generate a command that changes the position of the helicopter. In this case the cascade loop controller is activated and the controlled variables are $\lambda$ and $\epsilon$. The PID for travel generates the set point value for $\rho$.
- *IMU mode* this mode is accessed by performing the Fist gesture. In this case the horizontal and vertical motion of the test-bench are tied to the motions of the forearm of the user, for which the data of the inertial measurement unit (IMU) of the Myo Armband is acquired. In this mode, the cascade control loop is disconnected and the controlled variables become $\rho$ and $\epsilon$, and the set point value for pitch is directly sent from the PC.

### 6-DOF UR5 virtual manipulator

**Figure 8:**
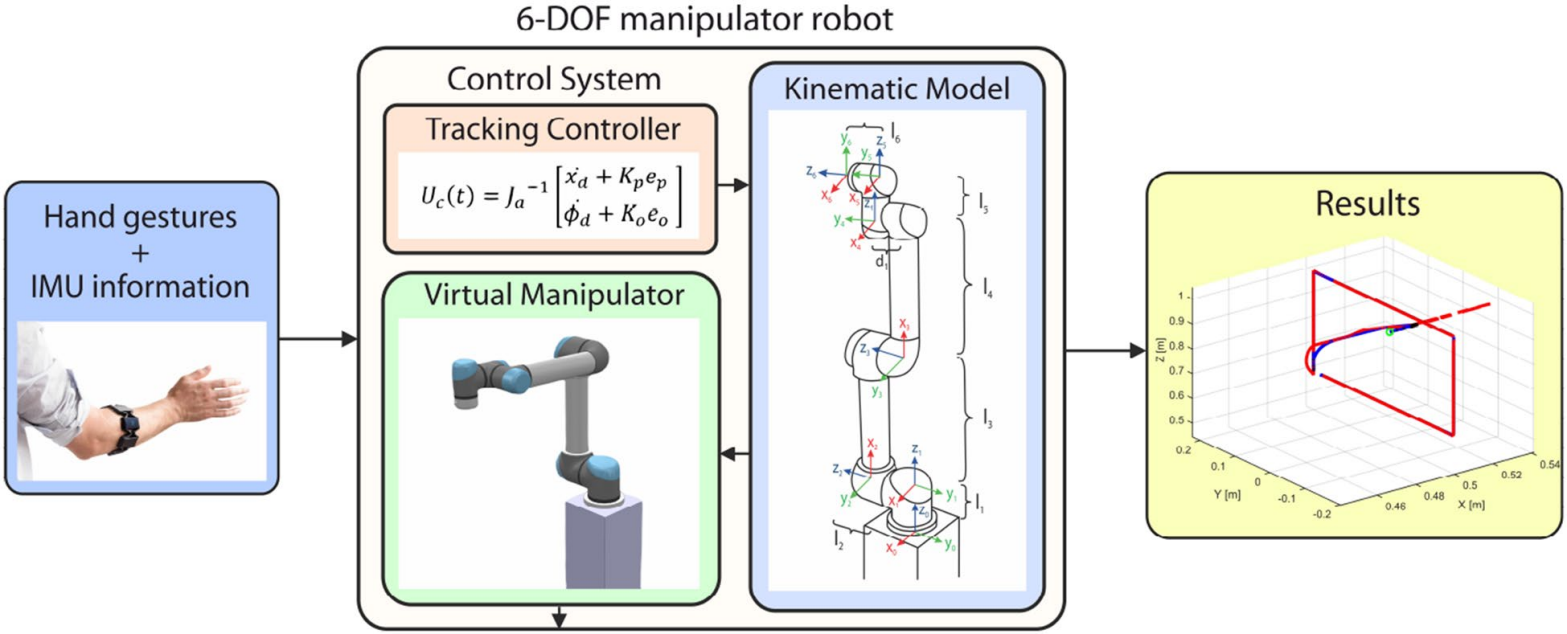
Architecture of the 6-DOF UR5 virtual manipulator controlled with the HGR system.

The UR5 robotic manipulator is a 6-DOF collaborative robot whose main characteristic is its agility due to its light weight, speed, and safety^36^. In this subsection, we describe each stage of the architecture proposed to control a virtual model of the UR5 robot, which is illustrated in Fig. 8. As can be observed, the proposed architecture is mainly conformed by a tracking controller, the UR5 kinematic model and its virtual model. We explain in detail each stage of the UR5 virtual manipulator control as follows.

- *Virtual Manipulator* A virtual environment was designed in CoppeliaSim robotics simulator, which is mainly focused on a virtual UR5 robotic manipulator that carries out painting tasks on a set of surfaces with strategic locations in order to demonstrate the usefulness of the HGR system and the tracking controller.
- *Tracking Controller* A PID control technique is used in order to achieve the main control objective which consists in the position and trajectory tracking of a UR5 virtual robot based on a desired position and orientation input reference.
- *Kinematic Model* The UR5 kinematic model describes the motion of the robotic manipulator regardless of forces and torques that originates it. The model used was obtained by the Denavit-Hartenberg (DH) standard method. Then, direct differentiation was applied in order to obtain the current state of the robot and contribute to the calculation process of the control actions by the tracking controller.

#### UR5 manipulator model

The UR5 robot manipulator movement can be defined by its kinematic and dynamic model. However, its dynamic model requires the study of forces and torques that originate its movement which is far harder to obtain in comparison with its kinematic model. Moreover, the dynamic model depends strongly on the mechanical structure of the robot, which by using a virtual robot, complicates the development of a control system. For that reason, our control system is designed taking into consideration mainly the kinematic model, which allowed us to obtain acceptable results at low speeds and smooth trajectory changes^37^. In order to obtain its kinematic model, it is necessary to obtain first its forward kinematic model obtained by the Denavit-Hartenberg (DH) standard method, which only uses the geometric properties of its structure, as in^38^. The forward kinematic model allows us to obtain the final end-effector position and orientation referenced to the robot’s base frame. Hence, the resulting coordinate transformation obtained by post-multiplication from the base frame to the end-effector of a 6-DoF robot manipulator is defined in Eq. (11) as follows:

11 $$\begin{aligned} {{T}}_{0}^{6}({{q}}) = {{T}}_{0}^{1}{{T}}_{1}^{2}{{T}}_{2}^{3}{{T}}_{3}^{4}{{T}}_{4}^{5}{{T}}_{5}^{6} \end{aligned}$$

The transformation matrix showed depends mainly on each joint configuration of the robotic manipulator where ${q}=[q_{1},\,q_{2},\,q_{3},\,q_{4},\,q_{5},\,q_{6}]^T$ and each joint position ${q}_{i}$ can have a value in the range from $-2\pi$ to $+2\pi$ (rad).

Once the forward kinematic model is obtained, then the Jacobian matrix of a robotic manipulator is calculated by direct differentiation. The resultant matrix is a function of the joint coordinates which allows us to find the speed of the end effector knowing the speed of each of its joints^39^. On the other hand, its inverse matrix is useful to find the speed that each joint requires in order to reach a specific speed for the end-effector^39^. Hence, the kinematic model of the UR5 is defined in Eq. (12) as follows:

12 $$\begin{aligned} {{\dot{h}}(t)} = \begin{bmatrix} {{\dot{x}}_e}(t)\\ {{\dot{\phi }}}(t) \end{bmatrix} = {J_{a}}({q}) {{\dot{q}}}(t) \end{aligned}$$

where $J_{a}$ is the analytical Jacobian matrix, ${{\dot{x}}_e}(t)=[{\dot{x}}(t),\, {\dot{y}}(t),\, {\dot{z}}(t)]^T$ contains the end-effector linear velocities, ${{\dot{\phi }}}(t)=[{\dot{\theta }}_r(t),\, {\dot{\theta }}_p(t),\, {\dot{\theta }}_y(t)]^T$ contains the time derivative of the Euler angles that has been chosen to define the end-effector orientation, and the variable ${\dot{q}}(t)$ in Eq. (13) is the input vector of the system that contains the joints velocities.

13 $$\begin{aligned} {{\dot{q}}}(t)=[{\dot{q}}_{1}(t),\, {\dot{q}}_{2}(t),\, {\dot{q}}_{3}(t),\, {\dot{q}}_{4}(t),\, {\dot{q}}_{5}(t),\, {\dot{q}}_{6}(t)]^T \end{aligned}$$

#### UR5 manipulator control system

**Figure 9:**
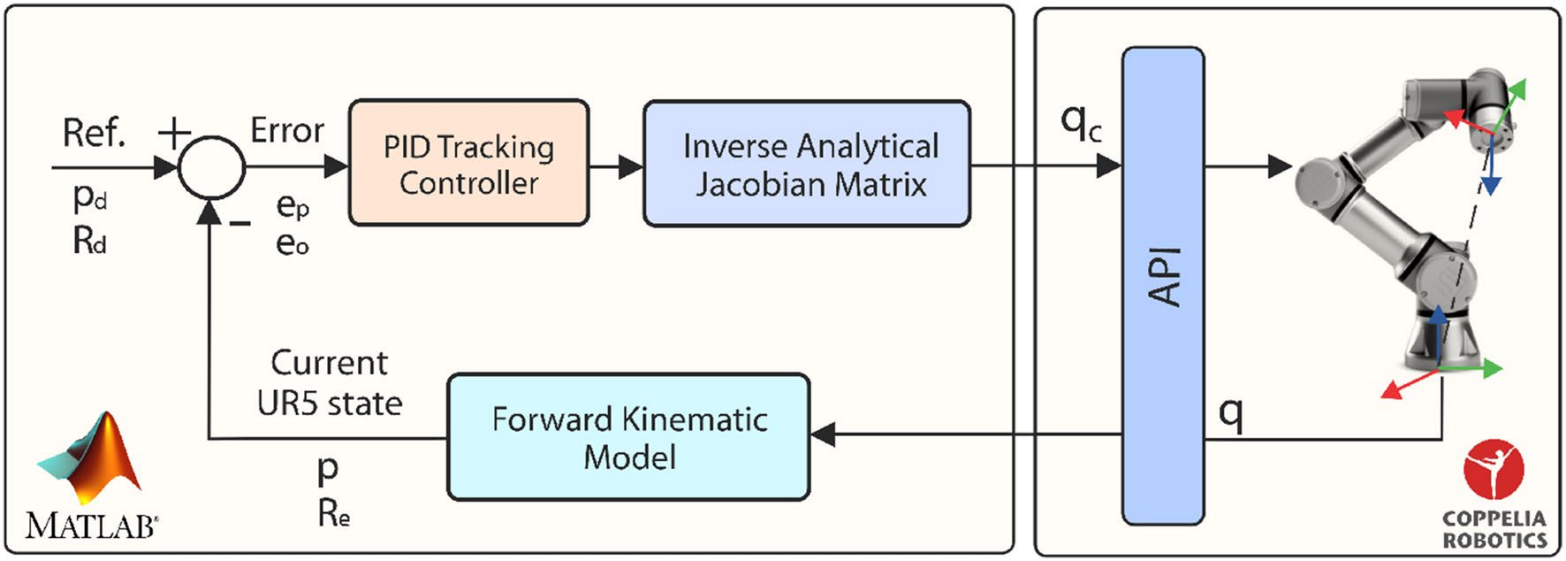
Control system proposed for a 6-DOF UR5 virtual manipulator.

In this subsection, we present the feedback control system to control a virtual UR5 robot, which is illustrated in Fig. 9. As can be observed, the proposal is conformed by the UR5 forward kinematic model and its Jacobian matrix. Futhermore, it also includes a minimum norm PID controller designed to control the UR5 virtual robot in CoppeliaSim, which is mainly based on its kinematic model. In addition, a Remote Application Programming Interface (API) was used in order to communicate both applications, Matlab and CoppeliaSim.

The control system proposed is divided into two main blocks, the first one is implemented in Matlab, and the second one in CoppeliaSim. In Matlab, the UR5 forward kinematic model is implemented in order to obtain its current state. This includes position and orientation of its end-effector, and subsequently, it allows us to obtain both, position and orientation error depending on the reference profile used as input of the control system^38^. In the same block, the inverse of the Jacobian matrix is also implemented since it constitutes a fundamental part of the proposal which enables the PID controller to compute the control actions^38^. On the other hand, in the CoppeliaSim block, we have the communication interface (API) and the virtual environment which includes the UR5 robot.

A minimum norm PID controller tries to minimize the control effort by finding an optimal solution through least squares approximations to achieve the control objective^40^. The controller is proposed in Eq. (14) as follows:

14 $$\begin{aligned} {q}_{c}(t) = {{J}_{a}^{-1}} \begin{bmatrix} \dot{x_d}+{K}_{p}{e}_{p} \\ {{\dot{\phi }}_d}+{K}_{o}{e}_{o} \end{bmatrix} \end{aligned}$$

where ${{J}}^{-1}$ is the analytical Jacobian inverse matrix of the robotic manipulator, ${{e}_{p}}$ and ${{e}_{o}}$ denote respectively the position and orientation errors, ${{\dot{x}}_{d}}$ and ${{\dot{\phi }}_{d}}$ are the time derivative of the end-effector desired position and orientation, respectively. ${{K}_{p}}$ and ${{K}_{o}}$ are positive definite matrices which contains the controller’s gain constants. These gain constants were obtained in order to minimize the Integrated Absolute Error (IAE) performance criterion, and as a result error is maintained close to zero.

The position error is given by ${e}_{p}={{p}_{d}}-{p}$, where ${p}_{d}$ and *p* denote respectively the desired and actual end-effector position. The orientation error ${e}_{o}$ definition depends on the orientation parameterization used, and it is defined in terms of the algebra of the rotation group and not of the vector algebra^41^. For that reason, we used an error definition which uses rotation matrices as follows: let us consider that $R_{e}$, ${R}_{d}$ describe the current and desired orientation of the UR5 end-effector with respect to the base frame, respectively. Therefore, the orientation matrix error is defined in Eq. (15) by expressing the relative orientation from $R_{e}$ to ${R}_{d}$ with respect to the base frame as follows^42^.

15 $$\begin{aligned} {{\tilde{R}}} = {{R}_{d}}{{R_{e}}}^{T} \end{aligned}$$

However, the use of this matrix in orientation control tasks is very limited due to the difficulty of handling its nine elements into the differential kinematics-based controller proposed in this work that uses a $6 \times 6$ Jacobian matrix. For that reason, it’s better to use an orientation $3 \times 1$ error vector, in terms of ${\tilde{R}}$. Therefore, in this paper, we use the following definition expressed in Eq. (16) which is proposed in^43^:

16 $$\begin{aligned} {e_o}({{\tilde{R}}}) = \frac{1}{2} \begin{bmatrix} {\tilde{r}}_{32}-{\tilde{r}}_{23} \\ {\tilde{r}}_{13}-{\tilde{r}}_{31} \\ {\tilde{r}}_{21}-{\tilde{r}}_{12} \\ \end{bmatrix} \end{aligned}$$

where ${\tilde{r}}_{ij}$ the *ij*-th element of ${\tilde{R}}$. Notice that ${e}_{o}({\tilde{R}})=0$ whenever $R_{e}={R}_{d}$.

It is worth noticing that the PID controller uses the analytical Jacobian instead of the geometric Jacobian *J* since it operates on error variables that are defined in the operational space of the robot which allows to describe the position and orientation to represent an end-effector task^42^. Both Jacobians are matrices that relate joint velocities with linear and angular velocities whose results are identical for linear velocities, but it’s different for angular velocities. The analytical Jacobian gives us the time derivative of the Euler angles that we chose to represent the orientation of the end effector. On the contrary, the geometric Jacobian gives us the angular velocities around *x*, *y* and *z* axes referenced to the robot base frame.

#### HGR for the 6-DOF UR5 manipulator

**Figure 10:**
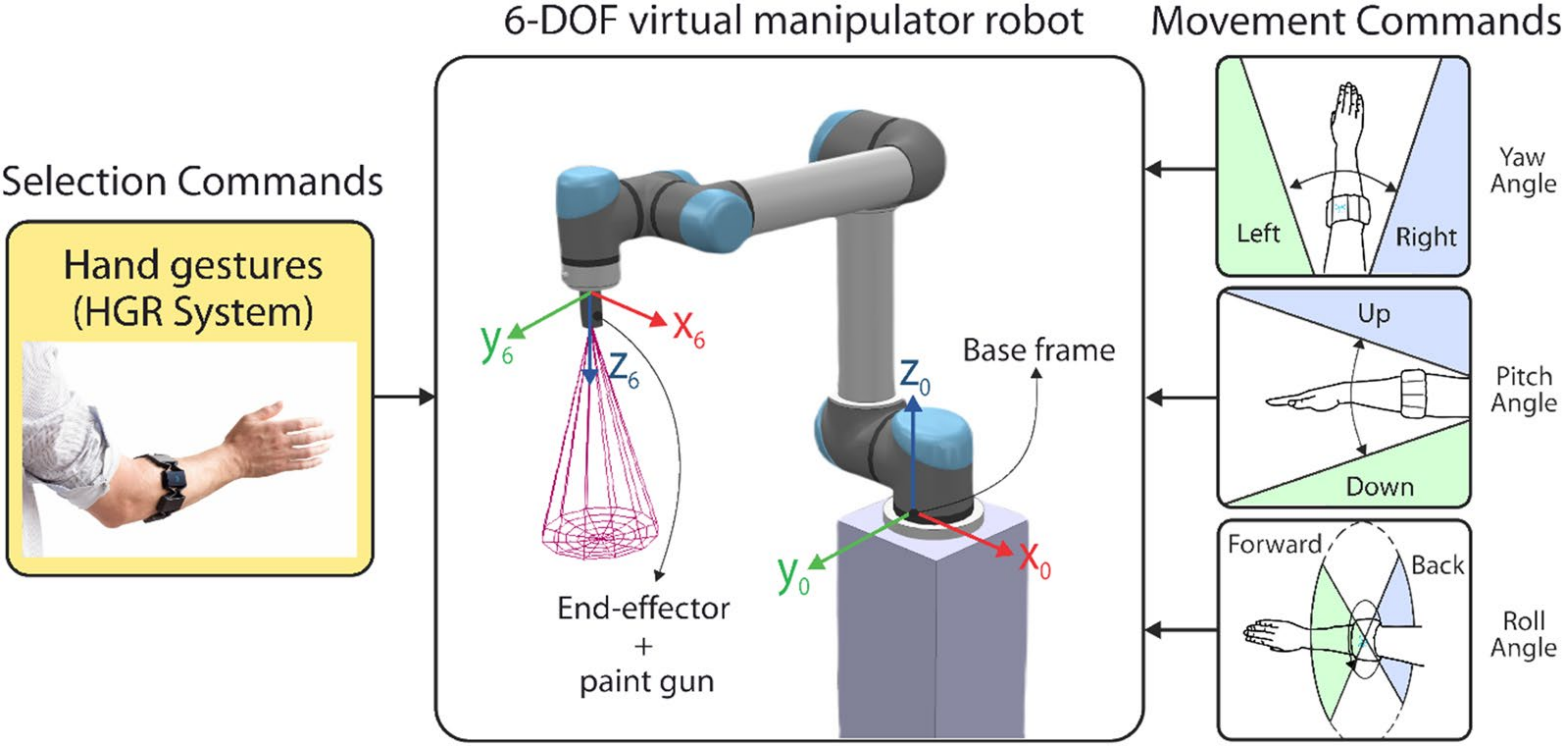
Movement and selection commands generated by the HGR system to manage the 6-DOF UR5 virtual manipulator^25,26^.

In order to show the HGR system effectiveness and fulfill the application control objectives, five different hand gestures are used. Selection commands are generated through hand gestures executions. On the other hand, Myo Armband’s IMU Data is used in order to adjust the position reference for the UR5 end-effector in the space as movement commands, as can be observed in Fig. 10. We explain in detail each command as follows.

**Figure 11:**
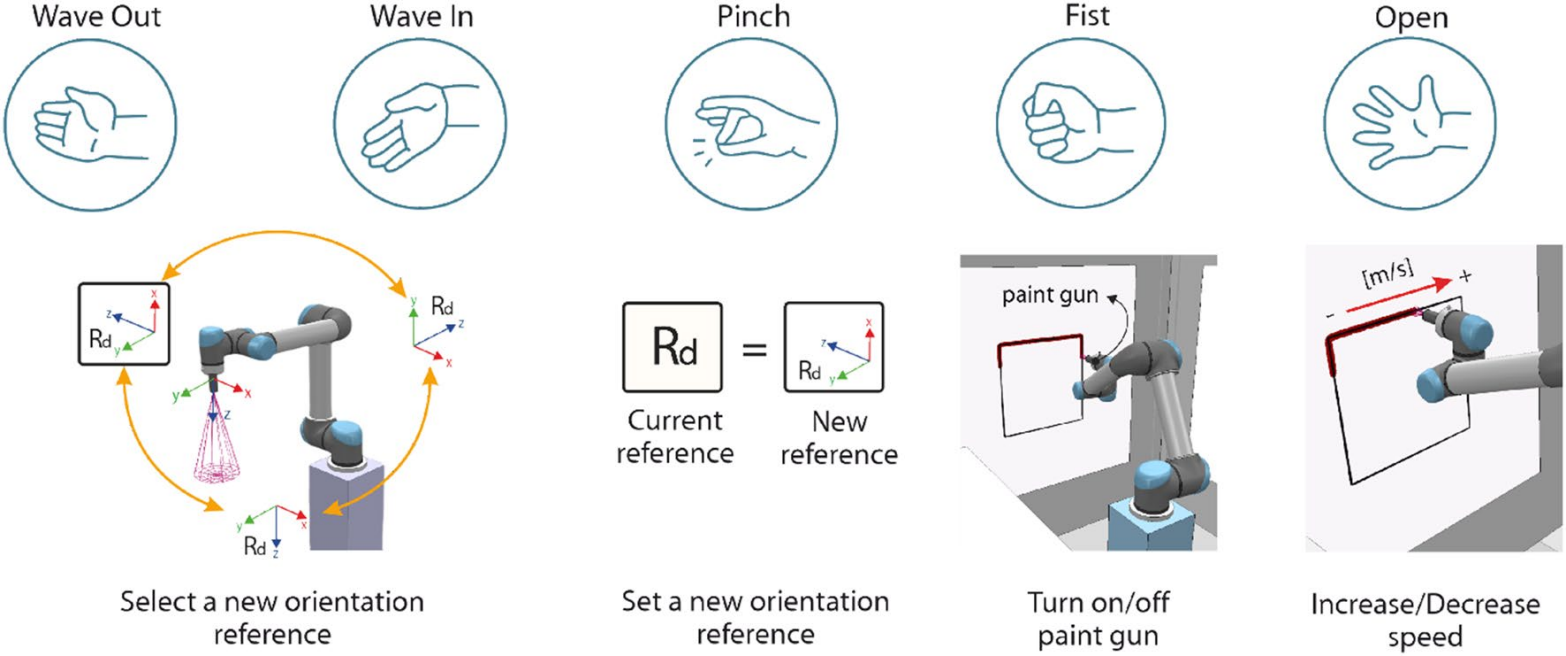
Mapping between hand gestures and selection commands for the 6-DOF UR5 Virtual Manipulator^25,26^.

- *Selection commands* Selection commands were selected for the UR5 robot in order for it to perform a specific action. They are generated once a hand gesture is executed and properly recognized by the HGR system, as can be observed in Fig. 11. We explain in detail the mapping between the hand gestures and selection commands as follows. A Fist gesture triggers the activation and deactivation of the paint gun attached to the UR5 end-effector. Wave in and Wave out gestures are used as commands to select a new orientation reference for the control system from a set of three different orientation matrices, which are predefined in accordance with the location of the surfaces established in the virtual environment. However, a new orientation reference is not set into the control system as the current orientation reference ${R}_{d}$ until we execute the Pinch gesture and gets recognized.
- *Movement commands* The UR5 can reach different locations as long as it is within its workspace. For that reason, the position reference in the space is established through motion commands generated by the Euler angles provided by the Myo Armband sensor. This movement commands change the position reference of the UR5’s end-effector, which are generated by setting an upper and lower limit to each Euler angle, as can be observed in Fig. 10. Therefore, the pitch angle allows moving the end-effector along the z-axis of the robot’s base frame whereas the yaw and roll angles allow changing the reference in its x and y axes, respectively. Basically, if any angle reaches its upper limit, the position reference will change causing the end-effector’s movement by the control system action in one direction, but if it reaches its lower limit, the end-effector will move in the opposite direction. If any upper or lower limit is not reached, the end-effector will remain immobile which also indicates a null position error. The movement along the axes once is generated, is uniform and it has a constant linear speed of 0.01 m/s. Finally, as an additional selection command, if we execute an open gesture and it is recognized by the HGR system the UR5 end-effector movement speed will increase from 0.01 m/s to 0.02 m/s or otherwise.

## Results

### HGR system results

In this subsection, we present the results EMG-IMU-based HGR system for the user-specific model trained. We first perform the validation procedure by finding the best possible hyper-parameters by evaluating data from 16 users. Then, we present the final testing results for 16 different users with the best hyper-parameters found during the validation procedure. The results of the validation and testing are explained as follows.

### Validation results

For the validation results, we trained and tested different models, which are based on the DQN algorithm that we present in previous sections. The best hyper-parameters found during the validation procedure are presented in Table 2. The validation results for the DQN-based model with the best hyper-parameters that we found were $95.7 \pm 3.34\%$ and $86.2 \pm 10.27\%$ for classification and recognition accuracy respectively.

**Table 2:** Best Hyper-parameters found during the validation procedure for the DQN algorithm.

Hyper-parameter name	Hyper-parameter values
Activation function between layers	Relu
Target Smooth Factor	$0.5e^{-3}$
Experience buffer length	60
Learn rate ($\alpha$)	$0.07e^{-3}$
Epsilon initial value	0.99
Epsilon greedy epsilon decay	$1.5e^{-4}$
Discount factor	0.99
Mini batch size	64
Optimizer	Adam
Gradient decay factor	0.85
L2 regularization factor	0.0003

### Testing results

**Figure 12:**
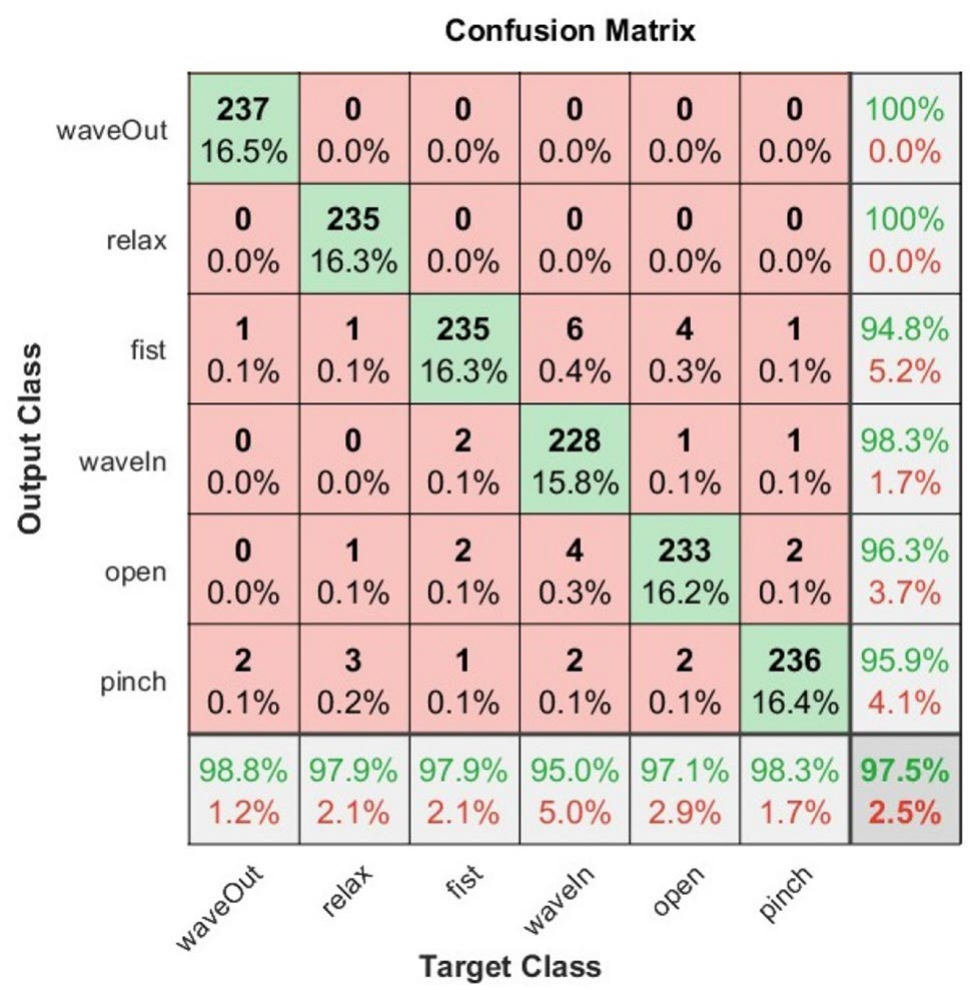
Confusion matrix with classification results for 16 user-specific HGR models of test set.

Based on the best hyper-parameters previously found during the validation procedure, we used the test-set composed of 16 different users to evaluate such user-specific models and obtain the testing results. This is useful to evaluate the best-found model with different data to analyze over-fitting. The test accuracy results for 16 users of the testing set were $97.45 \pm 1.02\%$ and $88.05 \pm 3.10\%$ for classification and recognition respectively. As can be noticed, there is barely a difference between the validation and the testing accuracy results, although the results are of different users. Thus, we can infer that our models are robust against over-fitting for the proposed data-set. Finally, we also present the confusion matrix that represents the classification results on the test set in Fig. 12, which allows us to observe in detail the results for each hand gesture. It is worth mentioning that the processing time of each window observation is on average 20 ms.

### Comparison with other works

In this subsection, we make a comparison between our proposed HGR method with other works of literature that use the six hand gestures used in this work and the Myo Armband sensor. This comparison is illustrated in Table 3, where it can be seen that our HGR method based on an RL algorithm that uses DQN and CNN-based agent representation is the one that obtains the best results.

**Table 3:** Obtained accuracy results of the proposed HGR model compared with other works.

Approach	Signal	Classification accuracy (%)
DQN and CNN-based agent (our RL method)	EMG-IMU	$97.45 \pm 1.02$
Q-learning and ANN (RL)^24^	EMG	90.78
SMV classifier with orientation correction (ML)^44^	EMG	92.4
SMV classifier with orientation correction (ML)^7^	EMG	94.9
SMV classifier (ML)^7^	EMG	81.2
K-NN with dynamic warping (ML)^20^	EMG	89.5
K-NN classifier (ML)^12^	EMG	86

### 3-DOF tandem helicopter test-bench control results

**Figure 13:**
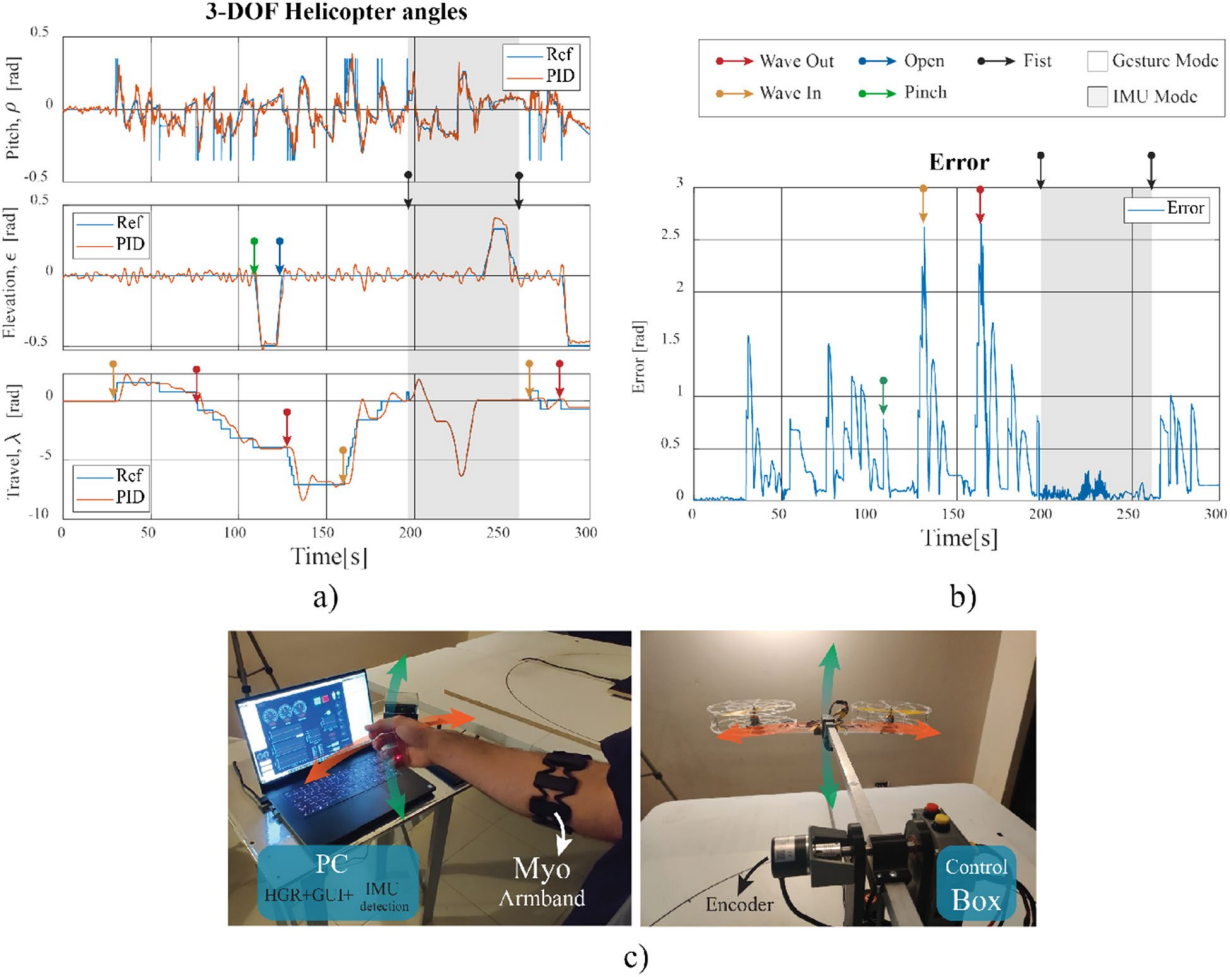
Response of the 3-DOF tandem helicopter test-bench used with the HGR system. (**a**) Time response graph for the three variables of the helicopter: pitch, elevation and travel. (**b**) Time response graph of the error. (**c**) Picture of the implemented prototype.

In this subsection, we present the results of the HGR system applied to control the 3-DOF tandem helicopter test-bench. In this test, the five different hand gestures were performed, and the behavior of the helicopter was recorded. As mentioned in previous sections, each gesture corresponds to a different command in which a reference value (or setpoint) is generated, and the variables of the helicopter test-bench must respond accordingly. In Fig. 13 the time response of the helicopter behavior is plotted. In Fig. 13a, the response of each variable is shown with the instants in which each gesture is performed (marked with different colored arrows). As it can be noted, each time the user makes a different gesture the setpoint value changes instantaneously, and the PID controller moves the helicopter in the corresponding direction. The PID controller can achieve a settling time of under 7 s and an overshoot of under 30% for the travel variable. The other variables have faster dynamics and therefore can achieve even lower settling times and overshoots.

In Fig. 13b, we show the error of the helicopter variables. To calculate this variable, the euclidean norm was used with the formula $e = \sqrt{{e_{\rho }}^2+{e_{\epsilon }}^2+{e_{\lambda }}^2}$, where $e_{\rho }, e_{\epsilon }$, and $e_{\lambda }$ correspond to the error of each of the three variables of the helicopter: pitch, elevation, and travel, respectively. As it can be seen, every time a gesture is performed, the error graph presents a peak and then the embedded PID controller on the 3-DOF helicopter regulates the error and rapidly approaches 0. It can also be noted that during IMU mode, the error remains in a low value, which is because in this operation mode the travel set point is equal to the measured output so that the pitch controller can act independently. This in turn generates an error in the travel variable, $e_{\lambda }=0$, and the total error *e* is also decreased.

From the error shown in Fig. 13b the mean squared error (MSE) was calculated to analyze the performance of the PID controller. In the case of the 3-DOF tandem helicopter, during the evaluation test, the mean squared error obtained was $MSE = 0.3438$. The formula shown in Eq. (17) was used to determine this value.

17 $$\begin{aligned} MSE = \frac{1}{N}\sum _{i=1}^{N} {e_{i}}^2 \end{aligned}$$

where *N* is the number of data points acquired during the test and $e_{i}$ is the value of the error during each discrete-time instant, *i*.

Finally, we can see in Fig. 13c a picture of the implemented prototype during the experiments, in which the HGR system was used to control the 3-DOF tandem helicopter test-bench. Additionally, a video demonstration of the helicopter in action is included in^45^.

### 6-DOF UR5 virtual manipulator control results

**Figure 14:**
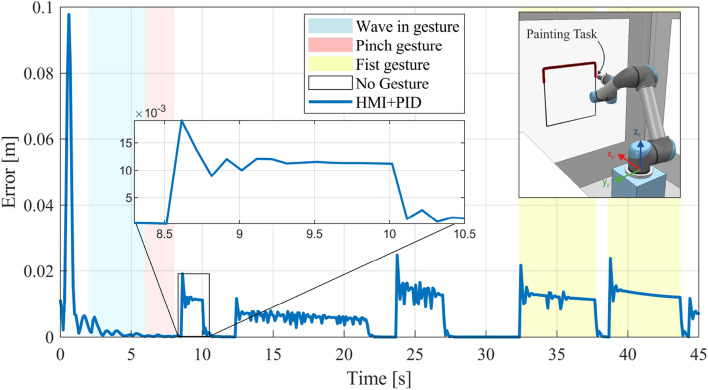
Position error evolution of the 6-DOF UR5 virtual manipulator used with the HGR system in order to achieve a square path tracking task during a painting task.

In this subsection, we present the results of the HGR system applied to control the 6-DOF UR5 virtual manipulator. For this, we perform a tracking and painting task in order to draw a square path through the selection and movement commands. The square path reference is located in the Y–Z plane in the virtual environment designed in CoppeliaSim. In Fig. 14, we present the position error ${e}_{p}$ evolution through its euclidean norm for the square path painting task highlighting by colored shades each period of time in which a hand gesture is performed.

**Figure 15:**
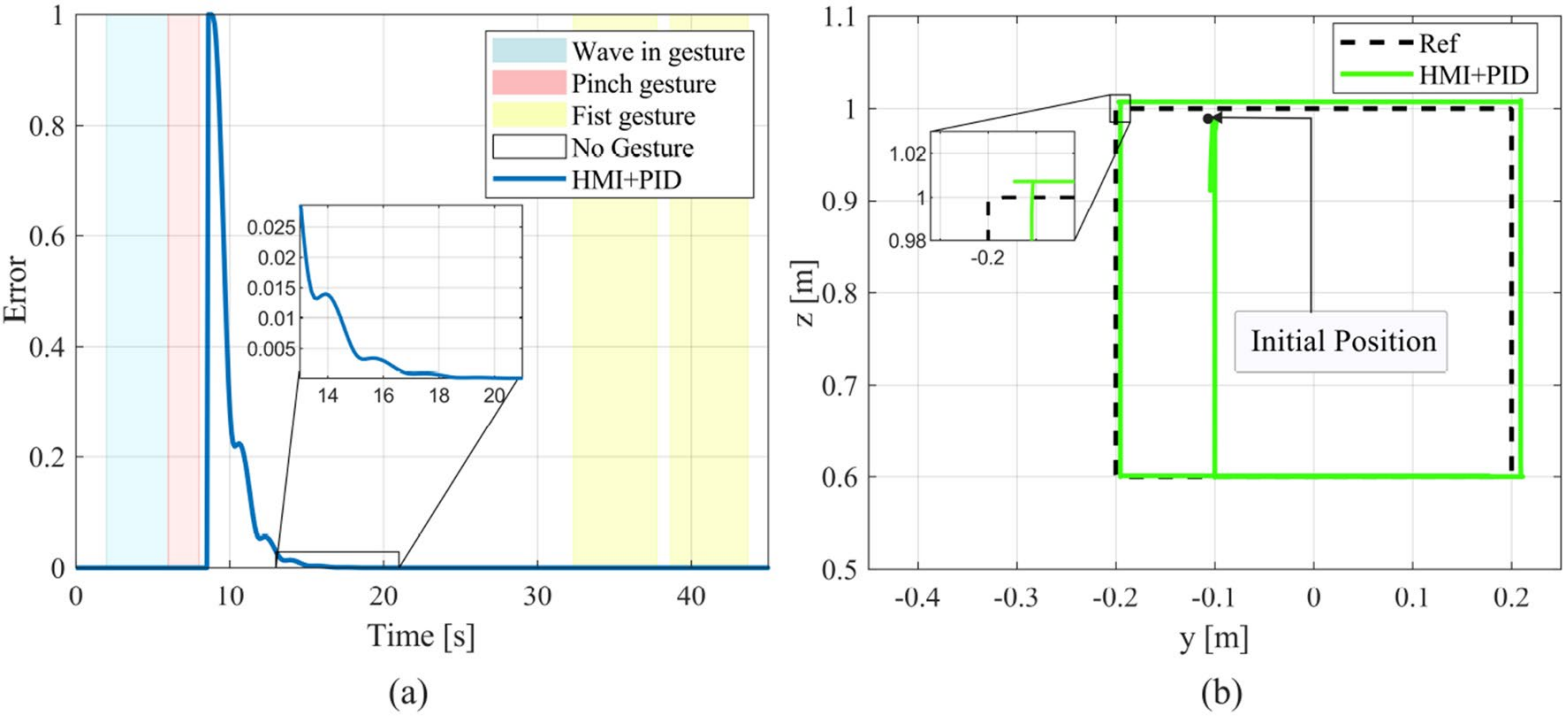
Square path tracking results (**a**) Evolution of the end-effector orientation error (**b**) 6-DOF UR5 virtual manipulator response in the Y–Z plane during the tracking task.

First, we performed a wave in gesture followed by a pinch gesture in order to aim the end-effector to the Y–Z plane by selecting a new orientation reference (or setpoint) from the available options. Subsequently, the end-effector is moved by movement commands from its starting point to a more adequate position close enough to the plane which allowed us to initiate the square path painting task by a fist gesture execution. As can be observed in Fig. 14, the position error remains close to zero in all axes, which means that the robot followed the square reference along its entire extension. Overall, the minimum norm PID controller presents acceptable robustness tracking the square path since it presents slightly abrupt reference changes in its corners, which cause small overshoots in the 6-DOF UR5 manipulator response that are way lower than 1%. Although the steady state error is null, the UR5 robot presents slight errors during the path tracking itself whose final impact on the painting task application is practically insignificant. In addition, in Fig. 15, we present the orientation error ${e}_{o}$ evolution for a square path tracking task and the complete system response in the Y–Z plane.

In Fig. 15a, we present the orientation error ${e}_{o}$ evolution which presents an abrupt change caused by the orientation reference transition generated by selection commands. As can be observed, the PID controller maintained the orientation error close to zero and achieved a settling time of under 5 (s) after the reference change.

We also tested the minimum norm PID controller within the control system structure through the mean squared error (MSE) performance criterion. Therefore, during the system evaluation, the mean squared error, obtained by Eq. (17) was $MSE = 0.0119$. A video of the system tests is available in^46^.

## Discussion

During the development of this work, a number of lessons were learned:

- The HGR classification and recognition obtained results indicate that using the DQN reinforcement learning algorithm to solve the sequential window classification problem of an EMG-IMU signal is possible. Additionally, the use of rewards for the successful classification of each sliding window, and the use of a reward for the recognition of an EMG-IMU sample, were successfully applied so that the agent-based on a CNN can work in the stage of classification of the HGR system.
- The validation procedure to find the best possible hyper-parameters for the development of the EMG-IMU-based HGR system was key to achieving high classification and recognition results. This procedure made it possible to increase the classification results up to $95\%$ for validation, which also helped to increase the recognition results. This, in turn, allowed obtaining up to $97\%$ classification for the testing procedure. Additionally, obtaining the testing and validation results with the best-obtained model helped us to compare their performance and evaluate over-fitting. This showed that the model’s performance in classification and recognition was similar for both data distributions by using the proposed dataset, which tells us that the model is robust to over-fitting.
- We demonstrated that both, the cascade PID and the minimum norm PID controllers, were able to reach acceptable robustness when controlling the 3-DOF tandem helicopter test-bench and the 6-DOF UR5 virtual manipulator respectively. This is because both controllers were able to reduce the error between the setpoint generated by the HGR system and the current position of the robot during the tracking tasks. Moreover, there were only slightly abrupt reference changes which cause small overshoots both methods present acceptable values of settling time and overshoot during the reference changes.
- Using the criterion of the mean square error (MSE) it was possible to compare the performance of the cascade and the minimum norm PID controllers applied to the 3-DOF tandem helicopter and the 6-DOF robot manipulator, respectively. From the obtained values it can be seen that the MSE calculated for the robot manipulator is considerably lower than that of the 3-DOF tandem helicopter test bench. This is due to the fact that in the case of the helicopter the results are acquired in a physical environment with an actual prototype and the error also reflects the effect of air turbulence, temperature, humidity, and other conditions. On the other hand, in the case of the 6-DOF UR5 manipulator, the proposed architecture was evaluated in a virtual environment through simulation. Additionally, the robot manipulator is a more stable platform than an aerial vehicle, in this case, represented by the 3-DOF tandem helicopter.
- The proposed integration of the EMG-IMU-based HGR system to control both a 3-DOF tandem helicopter test bench and a 6-DOF UR5 virtual manipulator through different sorts of commands obtained satisfactory results. The proposed method showed clear advantages for commanding the operation of different robotic platforms using hand gestures and arm movements for the generation of reference trajectories for the control system. In general, the system presented a low complexity and adaptability in terms of the requirements that each user needs to learn to use the proposed HGR interfaces to control both robotic platforms. However, although the learning process can be simple, its success and efficiency to achieve an objective depend largely on the user’s skills and experience, as well as the difficulty related to the application to be performed.

## Conclusions

In this work, we developed an EMG-IMU-based HGR system based on the DQN reinforcement learning algorithm for recognizing 6 different hand gestures. Then, we tested the effectiveness of the EMG-IMU-based HGR system and the IMU signals to control two different robotic platforms, a 3-DOF tandem helicopter test-bench, and a 6-DOF UR5 virtual manipulator. The results of $97.45 \pm 1.02\%$ and $88.05 \pm 3.10\%$ for classification and recognition respectively demonstrated to be sufficient to command the robotic platform’s operational modes. We also demonstrate the EMG and the IMU signals can be used to control the position and orientation of both robotic platforms, reaching low *MSE* values for both robotic platforms and acceptable values of settling time and overshoot. This work demonstrated that is possible to design a human-machine interface based on an EMG-IMU-based HGR system and IMU signals to successfully control the position, orientation, and operational modes of real and virtual robotic platforms. Future work will include different classification algorithms for delivering higher accuracy in the recognition of hand gestures, as well as different control strategies for the robotic platforms which might increase the system’s performance even further.

## Data Availability

The datasets generated and/or analysed during the current study are available in the EMG-IMU-EPN-100$+$ repository, https://laboratorio-ia.epn.edu.ec/en/resources/dataset/emg-imu-epn-100^27^.
